# The Great Pretender: Xanthogranulomatous Prostatitis Mimicking Prostate Cancer

**DOI:** 10.7759/cureus.86839

**Published:** 2025-06-27

**Authors:** Ashay A Patil, Amol Kamble, Ojas V Potdar, Shashank Sharma

**Affiliations:** 1 Urology, Grant Government Medical College and Sir JJ Group of Hospitals, Mumbai, IND

**Keywords:** benign prostatic hyperplasia, positron emission tomography with prostate-specific membrane antigen (psma pet), prostate cancer mimic, prostate imaging reporting and data system (pirad 5), transurethral resection of the prostate, xanthogranulomatous prostatitis

## Abstract

Xanthogranulomatous prostatitis (XGP) is a rare chronic inflammatory condition of the prostate that can clinically and radiologically mimic prostate cancer (PCa). We present a case of XGP in an elderly male with markedly elevated prostate-specific antigen (PSA) levels and imaging findings suggestive of locally advanced PCa. A 66-year-old male with a two-month history of intermittent fever was incidentally found to have two vesical calculi (13 mm and 9 mm) and grade 3 prostatomegaly (57 cc) with a large median lobe. He reported no lower urinary tract symptoms (LUTS). The patient underwent elective cystolithotripsy but developed hematuria, necessitating cystoscopic fulguration of the bladder neck. Postoperatively, his serum PSA was 100 ng/mL. Multiparametric MRI (mpMRI) revealed a Prostate Imaging Reporting and Data System (PIRADS) 5 lesion in the left transition zone with extraprostatic extension (stage T3b) and a PIRADS 2 lesion in the right peripheral zone. Ga-68 positron emission tomography with prostate-specific membrane antigen (PSMA PET-CT) demonstrated low-grade heterogeneous PSMA uptake (SUVmax 3.9) in the prostate. A transrectal ultrasound (TRUS)-guided prostate biopsy showed benign prostatic hyperplasia (BPH) with prostatitis. Following a failed voiding trial and recatheterization, the patient underwent transurethral resection of the prostate (TURP), which was uneventful. Histopathology revealed XGP with BPH. The patient had a successful postoperative recovery with effective voiding. XGP is an uncommon condition that can present with markedly elevated PSA and imaging findings suggestive of advanced PCa. This case underscores the importance of correlating histopathological findings with clinical and imaging data to avoid overtreatment.

## Introduction

Xanthogranulomatous prostatitis (XGP) is an exceedingly uncommon, benign inflammatory disorder of the prostate characterized histologically by sheets of lipid-laden (foamy) macrophages admixed with lymphoplasmacytic infiltrates and occasional multinucleated giant cells. Fewer than 60 well-documented cases have been reported worldwide, with the largest single-institution series describing only six patients; most affected men are in their sixth or seventh decade, although isolated cases have been reported as early as the fourth decade [1, 2]. The clinical picture is notoriously heterogeneous. While many patients present with lower urinary tract symptoms (LUTS) such as frequency, urgency, dysuria, or acute urinary retention, others are asymptomatic and come to attention because of unexplained fever or an incidental imaging abnormality. Serum prostate-specific antigen (PSA) may be normal, modestly elevated, or paradoxically reach levels (>100 ng/mL) typical of high-grade prostate cancer (PCa) [3].

Radiologically, XGP is a master mimic. On multiparametric MRI (mpMRI), it often produces low T2 signal, restricted diffusion, and early contrast enhancement, yielding a Prostate Imaging Reporting and Data System (PIRADS) score of 4 or 5. Positron emission tomography with prostate-specific membrane antigen (PSMA PET-CT) may show moderate tracer uptake (SUVmax ≈ 3-6), again overlapping with malignancy [4]. Because of this overlap, the differential diagnosis necessarily includes prostatic adenocarcinoma, nonspecific (idiopathic) granulomatous prostatitis, Bacillus Calmette-Guérin-induced granulomatous change, fungal or mycobacterial infection, malacoplakia, and, rarely, prostatic abscess or sarcoidosis [5]. Definitive distinction relies on histopathology obtained either from transrectal ultrasound-guided core biopsy or from surgical chips after transurethral resection of the prostate (TURP). Immunohistochemistry typically shows CD68-positive histiocytes and is negative for PSA or α-methyl-acyl-CoA racemase (AMACR), helping to exclude adenocarcinoma [6].

Management depends on symptom burden and the degree of obstructive pathology. Asymptomatic or mildly symptomatic patients with biopsy-proven XGP and no significant obstruction may be managed conservatively with antibiotics targeting common uropathogens and non-steroidal anti-inflammatory drugs; some authors have reported radiological regression on follow-up MRI [7]. Nevertheless, the majority require surgical intervention - most commonly TURP - to relieve bladder-outlet obstruction, eradicate niduses of infection, and obtain ample tissue for diagnosis. A simple open prostatectomy has been described for very large glands. Radical prostatectomy and radiotherapy are unwarranted once malignancy is excluded, yet several historical cases underwent such overtreatment before the histology was recognized [8].

Although the prognosis after appropriate surgery is excellent, delayed or missed diagnosis can lead to complications including recurrent urinary tract infection (UTI), chronic pelvic pain, persistent hematuria, prostatic abscess formation, and, in extreme cases, prostato-rectal or prostato-cutaneous fistulae [2, 4]. Awareness of XGP among urologists, radiologists, and pathologists is therefore paramount to prevent misdiagnosis and its attendant morbidity.

## Case presentation

**Figure 1:**
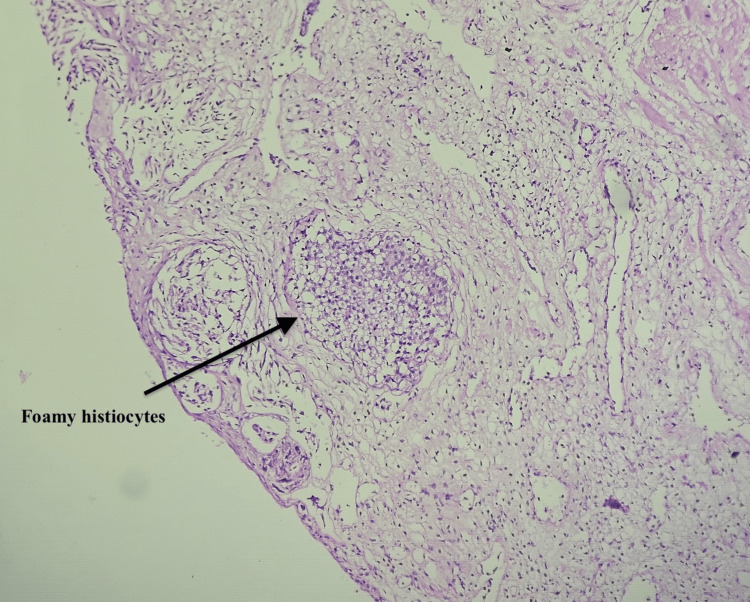
Microscopic examination showing foamy histiocytes with abundant pale-staining cytoplasm and eccentrically placed nuclei, characteristic of xanthogranulomatous prostatitis (H&E stain, 400×).

**Figure 2:**
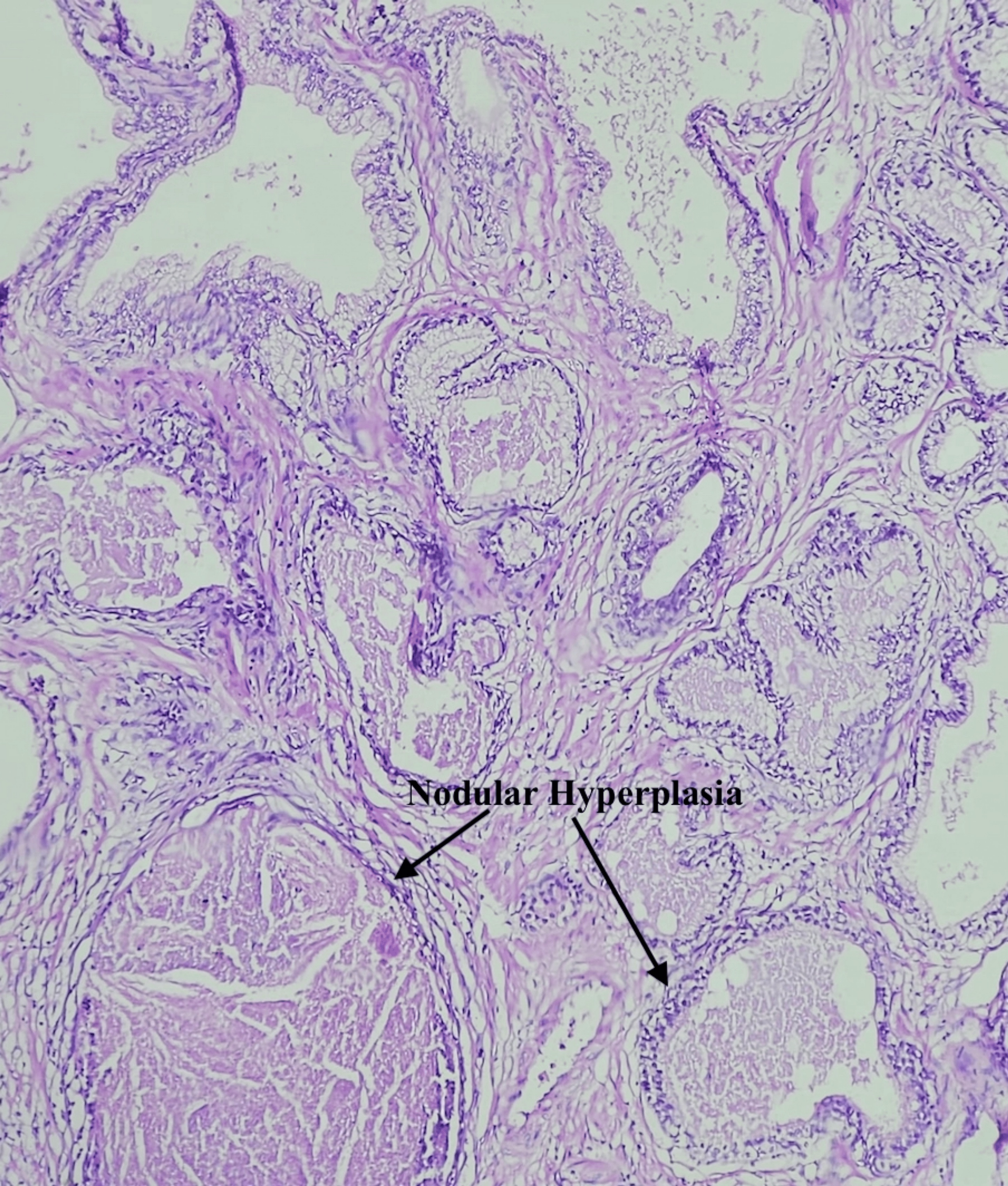
Nodular aggregates of histiocytes surrounded by inflammatory cells, including lymphocytes and plasma cells, within the prostatic stroma, consistent with xanthogranulomatous prostatitis (H&E stain, 200×).

XGP is a rare chronic inflammatory condition that can clinically and radiologically mimic prostate carcinoma, often resulting in unnecessary diagnostic and therapeutic interventions. We report the case of a 66-year-old male with no known comorbidities who presented with a two-month history of intermittent fever. During the evaluation, an incidental finding of two vesical calculi measuring 13 mm and 9 mm and grade 3 prostatomegaly with a prostate volume of 57 cc and a prominent median lobe was noted on ultrasonography. Notably, the patient did not report any LUTS. He underwent elective cystolithotripsy, which was uneventful. However, he subsequently developed hematuria requiring cystoscopic fulguration at the bladder neck. In the postoperative period, serum PSA was found to be markedly elevated at 100 ng/mL. Multiparametric magnetic resonance imaging (mpMRI) of the prostate revealed a PIRADS 5 lesion in the left transition zone with features of extraprostatic extension (T3b) and a PIRADS 2 lesion in the right peripheral zone. In view of the high suspicion of malignancy, a prostate-specific membrane antigen positron emission tomography-computed tomography (PSMA PET-CT) scan was performed, which showed a low-grade, heterogeneous PSMA-expressing lesion in the prostate with a maximum standardized uptake value (SUVmax) of 3.9 and scattered calcific foci. A transrectal ultrasound (TRUS)-guided prostate biopsy was subsequently carried out, which revealed benign prostatic hyperplasia (BPH) with prostatitis and no evidence of malignancy. Due to the trial void failure, the patient was recatheterized and planned for TURP. The TURP procedure was completed uneventfully, and the patient had a successful voiding trial postoperatively. Histopathological examination of the TURP specimen revealed features diagnostic of XGP along with BPH. Microscopy demonstrated numerous foamy histiocytes (Figure 1) and nodular aggregates of histiocytes accompanied by chronic inflammatory cell infiltrates (Figure 2).

## Discussion

XGP is an exceedingly rare subtype of granulomatous prostatitis, with fewer than 60 well-documented cases in the literature [1,2]. Its clinical significance lies in its capacity to closely mimic prostatic adenocarcinoma in both biochemical and imaging profiles. Patients may present with markedly elevated serum PSA, suspicious lesions on mpMRI, and even moderate radiotracer uptake on PSMA PET-CT, leading to a strong but misleading suspicion of high-risk PCa [3,4].

The pathogenesis of XGP remains incompletely understood, though proposed mechanisms include chronic or recurrent UTIs, obstructive uropathy (such as from bladder-outlet obstruction or stones), and prior urological instrumentation, all of which may trigger a localized xanthogranulomatous immune response. Histologically, this response is characterized by the presence of lipid-laden foamy macrophages, multinucleated giant cells, and lymphoplasmacytic infiltrates within the prostatic stroma [1,5].

Given the degree of clinical and radiologic overlap with malignancy, distinguishing XGP from prostate adenocarcinoma is critical to avoid overtreatment. While mpMRI and PSMA PET-CT are highly sensitive for detecting prostatic lesions, they are not specific for malignancy, as demonstrated in several reports of XGP exhibiting PIRADS 4 or 5 features and PSMA uptake in the absence of carcinoma [3,4,6]. Thus, definitive diagnosis hinges on histopathological examination, which remains the gold standard for differentiating benign granulomatous conditions from PCa [5,7].

Management of XGP is typically tailored to the patient’s symptom burden. In many cases, TURP is both diagnostic and therapeutic, offering relief of bladder-outlet obstruction while providing ample tissue for analysis. Open simple prostatectomy may be warranted in cases with gross prostatic enlargement [2,4,8]. Most patients respond well to surgical treatment, and recurrence is rare.

This case underscores the importance of maintaining a broad differential when encountering elevated PSA and PIRADS 5 lesions, particularly in the setting of prior instrumentation or infection. Prompt histopathological evaluation can prevent misdiagnosis, reduce patient morbidity, and avoid unnecessary radical therapies. Greater awareness of XGP among urologists, pathologists, and radiologists will help reduce the risk of overtreatment in similar presentations.

## Conclusions

XGP is a rare benign inflammatory condition that can clinically, biochemically, and radiologically mimic aggressive prostate cancer. This case emphasizes the critical importance of correlating histopathological findings with clinical presentation and advanced imaging to avoid unnecessary radical interventions. Increased awareness and recognition of XGP among urologists, radiologists, and pathologists are essential to ensure accurate diagnosis and appropriate management, thereby preventing overtreatment and its associated morbidity.
